# A national epidemiological study investigating risk factors for police interrogation and false confession among juveniles and young persons

**DOI:** 10.1007/s00127-015-1145-8

**Published:** 2015-11-04

**Authors:** Gisli H. Gudjonsson, Jon Fridrik Sigurdsson, Inga Dora Sigfusdottir, Bryndis Bjork Asgeirsdottir, Rafael A. González, Susan Young

**Affiliations:** King’s College London, London, UK; Reykjavik University, Reykjavík, Iceland; University of Iceland and Landspitali-The National University Hospital of Iceland, Reykjavík, Iceland; Imperial College London, London, UK; University of Puerto Rico Graduate School of Public Health, San Juan, USA; Broadmoor Hospital, WLMHT, London, UK; Faculty of Medicine, Centre for Mental Health, Imperial College London, 7th Floor Commonwealth Building, Du Cane Road, London, W12 0NN UK

**Keywords:** Epidemiology, Interrogation, False confessions, ADHD, Conduct disorder, Offending

## Abstract

**Purpose:**

The principal aims of this study are to identify risk factors associated with police arrest and false confessions and to investigate whether the severity of the ADHD condition/symptoms increases the risk.

**Methods:**

22,226 young persons in Iceland anonymously completed self-report questionnaires screening for conduct disorder and ADHD. In addition, they stated whether they had a diagnosis of ADHD and had received ADHD medication, and their history of offending, police interrogation and false confession. Participants were stratified into two age groups, 14–16 and 17–24 years.

**Results:**

The older group was significantly more likely to have been interrogated by the police but the younger group were much more vulnerable to false confession during interrogation. Males were more likely to be at risk for both than females. The severity of the ADHD condition increased the risk of both interrogation and false confession. Negative binomial regressions showed that age, gender, conduct disorder, offending, and ADHD symptoms were all significant predictors of both interrogations and number of false confessions. Conduct disorder was the single best predictor of police interrogation, but the findings were more mixed regarding false confessions. Young people presenting with a combination of severe ADHD and comorbid conduct disorder had the worst outcome for both interrogation and false confessions.

**Conclusions:**

The findings endorse the need for support of persons with ADHD to be put in place to ensure fair due process and to prevent miscarriages of justice.

## Introduction

It is important to identify risk factors associated with police interrogation and false confessions in order that appropriate safeguards may be applied. Age is an important predictor of outcome. A review of the literature revealed that juveniles are more vulnerable to giving a false confession during interrogation than adults [1–5]. The extent of offending behaviour (OB) is also predictive of false confessions both among juveniles in community samples [6, 7] and adult prisoners [8]. Males are more likely to be interrogated than females [6], but uncertainties remain regarding the gender risk for giving a false confession due to the small number of female false confession participants in previous studies. The large sample in the current study should give a definitive answer regarding the role of both age and gender in interrogations and false confessions.

Given the high rates of ADHD reported in the prison population of 26 % for adults and 30 % for youths [9], it is often assumed that ADHD is a salient risk factor for offending. The risk, however, may be more attributed to conduct disorder (CD; or antisocial personality disorder in an older group) due to the irresponsible life style and disregard for the consequences of behaviour associated with these disorders [10]. CD and ADHD are associated conditions [11–13] with a common genetic influence [14]. Young and Gudjonsson [15] have demonstrated the importance of antisocial personality traits in people clinically diagnosed with ADHD, which makes them susceptible to social maladjustment and delinquency. Lynam [16] suggests that children with a combination of ADHD and CD are at greatest risk of becoming persistent offenders. Recent research has suggested that the relationship between ADHD and offending is largely mediated by conduct disorder (CD), substance misuse and association with delinquent peers [17]. It is likely that those mediating factors bring them to the attention of police rather than their ADHD symptoms per se, whereas their ADHD may leave them additionally vulnerable to falsely confessing to a crime during interrogation.

Previous work has not attempted to tease out the relationship between predictors of arrests and false confessions. Frequency of arrests has been predicted by both an ADHD diagnosis and childhood CD symptoms, the latter being the more powerful predictor than the former [18]. Loeber, Burke, Lahey, Winters, & Zera [19] found that CD is a major risk factor in terms of later criminal behaviour and police arrests. Satterfield et al. [20] conducted a 30-year follow-up of 179 clinically referred and treated hyperactive boys and 75 controls, assessed in childhood when aged 6–12 years. A high rate of adult arrests (44.1 %) was found in contrast to controls (14.7 %), with the ADHD group being 4.57 times more likely to be arrested than controls, 4.68 more likely to be convicted and 4.08 times more likely to be incarcerated. The highest rate of arrest (59 %) was between the ages of 18–21 and the rate declined with age. Most of the sample (78 %) had childhood behavioural symptoms consistent with CD. Langley et al. [21] in a 5-year follow-up of 126 children diagnosed with ADHD and treated in childhood (mean age 9.4 years), found that 61 % reported at least one police contact at follow-up (mean age 14.5 years). Police contact in the previous 3 months was 27 %. At follow-up, 31.5 % were diagnosed with CD and 63 % were currently prescribed stimulant medication.

So far as false confessions are concerned, Gudjonsson, Sigurdsson, Bragason, Einarsson, & Valdimarsdottir [22] found that antisocial personality traits and the extent of general offending were highly predictive of false confessions among college and university students. In a prison sample, Gudjonsson, Sigurdsson, Einarsson, Bragason, & Newton [23] reported that 41 % of prisoners who were symptomatic for ADHD had a history of false confession in contrast to 18 % of non-ADHD prison controls. ADHD was found to predict false confessions above antisocial personality disorder [24] suggesting that the high rate of false confessions reported among the ADHD group was not significantly mediated by their antisocial personality disorder. An epidemiological study reported a similar finding [11].

What has not been systematically investigated for age, gender, ADHD, CD and OB is whether different factors pose a risk for being arrested for the purpose of interrogation and false confessions. The present study aimed to address this gap in knowledge by conducting a study with definitive power to investigate whether the pattern of risk for juveniles and young persons to be arrested and interrogated by the police for suspected crimes is similar to the risk that they will give a false confession. The literature reviewed suggests that the severity of ADHD symptoms is likely to be an important mediator of outcome, thus the present study included measures of severity.

CD and OB are likely to be salient triggers for arrest and interrogation, whereas ADHD symptoms per se are probably less important at the point of arrest. In contrast, once arrested and interrogated ADHD symptoms are likely to become more relevant to how they cope with interrogation in terms of the risk of giving a false confession [24].

The current sample was stratified into two age groups, 14–16 and 17–24 years. This categorisation is based on the UK Police and Criminal Evidence Act 1984 (PACE) Code of Practice where those below the age of 17 are classified as ‘juveniles’ and require the services of an ‘Appropriate Adult’ during police interviews. The role of an ‘Appropriate Adult’ is to provide support and advice to people with intellectual disability and other mental health difficulties and is the main protection for juveniles and ‘mentally vulnerable’ detainees during interviews by police [25].

Hypothesis 1 is that older youth (17–24) are more likely to be interrogated by police than juveniles (14–16), whereas the younger group is likely to report more instances of false confessions during interrogation. Hypothesis 2 is that males are more likely to be interrogated than females and when interrogated they are more likely to give false confessions. Hypothesis 3 is that CD and OB are better predictors of interrogation than false confessions, whereas ADHD is a better predictor of false confessions than CD and OB. Hypothesis 4 is that the severity of the ADHD condition/symptoms increases the risk for police interrogation and false confession.

## Method

### Participants

The sample was comprised of 22,226 young persons in Iceland; 10,838 (48.8 %) were in the final 3 years of mandatory education and 11,388 (51.2 %) were in further education at college. The mean age for the total sample (496 did not give their age) was 16.4 years (SD = 1.9; range 14–24 years). There were 10,778 males (49 %) and 11,211 females (237 did not give their gender). For the purpose of analysis, the participants were categorised into two age groups, those aged 14–16 (*n* = 13,933) and 17–24 (*n* = 7797).

144 schools of mandatory education in Iceland were represented in the study. The current sample included 86 % of all mandatory students in Iceland at the time of data collection which took place in February 2012. In Iceland, 95 % of those who finish mandatory education go into further education in colleges. With regard to the college students, all 40 colleges of further education in Iceland were represented. The current sample included 70.5 % of all students registered in the colleges at the time of the data collection, which took place at the end of 2010 and beginning of 2011. We have no information on those students who did not participate in the Survey. The students who did not complete the survey were primarily those who did not turn up for scheduled class on the day of the Survey.

All the schools and pupils consented to take part in the survey. Approval was provided by the Icelandic Ministry of Education and the survey was conducted in accordance with the Icelandic Science Ethics Committee ethical code of conduct, as well as national law. 

#### Measures

A detailed survey questionnaire asked about participants’ family circumstances, education, mental health problems, offending, police involvement and false confession [11].

The survey measures included the following:

*Barkley Current Symptoms Scale (BCS)* [26]. This scale corresponds with DSM-IV criteria for ADHD symptoms. Each of the 18 items, nine items relating to inattention and nine items to hyperactivity/impulsivity, is scored on a 4-point rating scale for frequency of symptoms experienced during the previous 6 months. Scores range between 0 and 27 for each of the two subscales (Inattention and Hyperactivity/Impulsivity) and 0–54 for the Total scale. In the current study, a screening diagnosis for ADHD symptoms was obtained if six or more of the inattention or hyperactivity/impulsivity items were endorsed as either ‘often’ or ‘very often’ (i.e. 6 out of the 9 items had to be endorsed on either subscale). This is the scoring criterion used in previous research [11, 24, 27].

*Questions about ADHD diagnosis and medication*. Participants were specifically asked ‘Have you been diagnosed with ADHD?’ and ‘Are you currently taking medication for ADHD?’. Both answers were endorsed as either ‘Yes’ or ‘No’.

*Severity of ADHD*. Two measures of severity were obtained from (a) categorising the symptomatic group into predominantly inattentive, predominantly hyperactive/impulsive, and combined type; the combined type represents greatest severity (see Tables 1, 2); and (b) combining those who are currently self-reporting ADHD symptoms and to be receiving ADHD medication (which implies that the medication may not be fully effective in reducing symptoms below screening diagnostic threshold). These are different but overlapping measures of severity. In the latter case this resulted in a hierarchy of presentation according to severity of symptoms as follows, which takes into consideration both current ADHD symptoms and medication status (see Table 4):

**Table 1:** Differences in the predictor variables between those interrogated and those not interrogated

	Interrogated *N* (%)	Not interrogated *N* (%)	*χ* ^2^ *df* = 1	OR (95 % CI)
ADHD-symptomatic	340 (11.4)	698 (3.8)	316.2*	3.1 (2.8–3.7)
ADHD-inattentive	136 (4.6)	313 (1.7)	100.2*	2.7 (2.2–3.4)
ADHD-hyperactive	71 (2.4)	189 (1.0)	38.3*	2.3 (1.6–3.1)
ADHD-combined	133 (4.5)	196 (1.1)	192.5*	4.3 (3.4–5.4)
Current medication	290 (9.9)	636 (3.5)	245.8*	3.0 (2.6–3.5)
History of diagnosis	697 (23.3)	1564 (8.6)	566.8*	3.2 (2.9–3.5)
Conduct disorder	1153 (39.4)	1791 (9.9)	1818.4*	5.9 (5.4–6.4)
Offending behaviour	1187 (40.8)	2993 (16.6)	919.7*	3.4 (3.2–3.8)

* *p* < 0.001

**Table 2:** Differences in the predictor variables between those giving a false confession and those with no history of a false confession

	False confession *N* (%)	No false confession *N* (%)	*χ* ^2^ *df* = 1	OR (95 % CI)
ADHD-symptomatic	95 (21.9)	241 (9.6)	55.4*	2.6 (2.0–3.4)
ADHD-inattentive	26 (6.0)	109 (4.3)	2.3	1.4 (0.9–2.2)
ADHD-hyperactive	20 (4.6)	49 (2.0)	11.4*	2.4 (1.4–4.1)
ADHD-combined	49 (11.3)	83 (3.3)	55.2*	3.7 (2.6–5.4)
Current medication	99 (24.1)	188 (7.6)	108.1*	3.9 (3.0–5.1)
History of diagnosis	167 (40.5)	505 (20.4)	80.2*	2.7 (2.1–3.3)
Conduct disorder	246 (59.6)	893 (36.1)	81.8*	2.6 (2.1–3.2)
Offending behaviour	239 (58.0)	943 (38.1)	57.6*	2.2 (1.8–2.8)

* *p* < 0.001

Severity 1: not on medication and not meeting screening diagnosis on BCS (*N* = 19,492; 91.4 %)

Severity 2: not on medication but meeting screening diagnosis on BCS (*N* = 868; 4.1 %)

Severity 3: currently on medication but not meeting screening diagnosis on BCS (*N* = 791; 3.7 %)

Severity 4: currently on medication and meeting screening diagnosis on BCS (*N* = 179; 0.8 %)

*The oregon adolescent depression project conduct disorder screen (OADP-CDS)* [28]. This 6-item self-report screen of adolescent conduct behaviours, rated on a 4-point Likert scale, provided a total score ranging between 6 (no endorsement of any behaviour) and 24 (maximum endorsement of each behaviour). The OADP-CDS has been shown to have good internal consistency, test–retest reliability, and good screening efficiency for detecting lifetime conduct disorder [28]. A cutoff score of 10 or higher was used as an indicator of the presence of conduct disorder.

*Offending behaviour(OB)* [29]. This five-item scale measures the extent of self-reported offending. The question asked is: “How often have you done the following?” and five delinquent behaviours are rated (e.g. minor theft, major theft, violence, vandalism and burglary) during the previous 12 months. Answers range from 1 (never) to 7 (18 times or more). We dichotomised the group categorically: a score of 1 (no offending) versus a score of 2 or higher (offending).

*Police interrogationand confessions questionnaire* [11]. Participants were asked about their experiences of police interrogation, confessions and false confessions as follows:

‘How often have you been interrogated at a police station as a suspect in a criminal offence?’ and ‘Have you ever confessed during police interrogation to a criminal offence that you did not commit (i.e. you had nothing to do with the offence and are completely innocent)?’ Replies were rated on the five-point scale: ‘Never’, ‘Once’, ‘Twice’, ‘3–5 times’, ‘6 or more times’.

Because in the authors’ previous experience participants have been reluctant to specify a precise number for these variables, thereby leaving a great deal of missing data, these otherwise count variables were coded in ordered categories.

### Procedure

The participants were approached by teachers in scheduled classes and invited to participate in the survey. The participants were assured that their answers would be anonymous. The questionnaire took up to 80 min to complete and upon completion the students sealed them in a blank envelope and left it by the exit of the classroom.

### Analytical strategy

Frequencies were reported for all categorical variables, and means with their standard deviations for continuous descriptive variables.

To establish independence in the proportions of the observations of all binary and categorical variables, we used Chi-square (χ^2^) tests. For all these binary association tests, odds ratios (OR) with 95 % confidence intervals were calculated as a measurement of their effect size.

Taking into account the proportion of zero responses, and their overall distribution we treated the response variables number of interrogations and of false confessions as count, and fitted negative binomial regression (NBR) models. A Poisson distribution is appropriate in accounting for observed heterogeneity when using count data but is not when there is overdispersion, as observed in both these response variables. And although there was a high proportion of zero responses on both variables, a zero-inflated model presupposes the existence of two zero processes (i.e. two plausible reasons why there are zero responses) [30]. In our context, NBR was the most appropriate. For each multivariate model of number of interrogations and number of false confessions, the following variables we entered simultaneously: age group (<17, 17 or more), gender, CD, OB and ADHD-symptomatic.

Model beta coefficients were exponentiated, with odds ratios (OR) as indicators of the magnitude of associations in binary outcome models, and incidence rate ratios (IRR) in negative binomial regressions. A significance level of *α* < 0.05 was adopted throughout. All analyses were performed using Stata version 13 [31].

## Results

### Base rates of interrogation and false confession

Out of 21,260 participants where data were available, 2987 (14.0 %) reported having been interrogated at a police station. Of those, 1739 (58.2 %) had been interrogated only once, 586 (19.6 %) twice, 408 (13.7 %) three to five times, and 254 (8.5 %) six or more times. Males were significantly more likely to report having been ever interrogated than females, 19.8 and 8.7 %, respectively (*χ*^2^ = 542.2, *df* = 1, *p* < 0.001, OR = 2.6, 95 % CI 2.4–2.8), as well as those in the older age group (21.3 versus 10.1 %; *χ*^2^ = 369.5, *df* = 1, *p* < 0.001, OR = 2.2, 95 % CI 2.0–2.4).

As far as the reporting of false confessions is concerned, of those 2947 participants interrogated where data on false confessions were available, 434 (14.7 %) reported having made a false confession; out of those, 264 (60.8 %) had made a false confession once, 82 (18.9 %) made a false confession twice, 40 (9.2 %) three to five times, and 48 (11.1 %) six or more times. Males were significantly more likely to report having made a false confession than females, 16.2 and 11.4 %, respectively (*χ*^2^ = 11.7, *df* = 1, *p* < 0.001; OR = 1.5, 95 % CI 1.2–1.9). Those in the younger age group (20.0 %) were more likely than those in the older group (10.3 %) to report having given a false confession (*χ*^2^ = 50.9, *df* = 1, *p* < 0.001, OR = 2.2, 95 % CI 1.7–2.7).

### Base rate for predictors of vulnerability

Out of 22,226 participants, 1097 (4.9 %) met screening criteria for self-reported ADHD symptoms, of whom 469 (2.1 %) were predominantly inattentive type, 273 (1.2 %) hyperactive/impulsive type, and 353 (1.6 %) were combined type. 2288 (10.8 %) reported having received a diagnosis of ADHD and 946 (4.5 %) reported to be currently taking medication for ADHD. The great majority (76.8 %) of those of the comorbid type reported not being currently on ADHD medication. Of the total sample, 3098 (14.3 %) met screening criteria for CD and 4207 (20 %) reported having committed one or more offences.

Significantly more males than females were ADHD combined type, 1.8 and 1.4 %, respectively (*χ*^2^ = 6.9, *df* = 1, *p* < 0.05, OR = 1.3, 95 % CI 1.1–1.6); currently taking ADHD medication, 6.2 and 2.9 % (*χ*^2^ = 134.0, *df* = 1, *p* < 0.001, OR = 2.2, 95 % CI 1.9–2.5); reported having received a diagnosis of ADHD, 13.7 % and 8.2 (*χ*^2^ = 166.4, *df* = 1, *p* < 0.001, OR = 1.8, 95 % CI 1.6–1.9); classified as CD, 19.4 and 9.5 % (*χ*^2^ = 425.3, *p* < 0.001, *df* = 1, OR = 2.3, 95 % CI 2.1–2.5); and had committed offences, 25.2 and 15.2 % (*χ*^2^ = 325.4, *df* = 1, *p* < 0.001, OR = 1.9, 95 % CI 1.8–2.0).

### Risk predictors of police interrogation and false confessions

Table 1 shows the relationship between being interrogated by the police and the risk predictor variables (ADHD symptoms, current ADHD medication, history of ADHD diagnosis, conduct disorder, offending behaviour). All predictors were significant for interrogation by police, the largest effect size being found for CD (OR = 5.9, 95 % CI 5.4–6.4) and ADHD-combined (OR = 4.3, 95 % CI 3.4–5.4), followed by OB (OR = 3.4, 95 % CI 3.2–3.8). Table 2 shows that for false confessions, the strongest predictors were the ADHD measures, particularly being currently on medication (OR = 3.9, 95 % CI 3.0–5.1) and an ADHD-combined classification (OR = 3.7, 95 % CI 2.6–5.4).

### Models for number of interrogations and false confessions

Table 3 summarises the outcome of the negative binomial regression models for number of interrogations and false confessions. The predictors entered were: Age group (i.e. 14–16 versus 17–24), gender (male = 1, female = 2); CD, OB and ADHD-symptomatic. A forced entry method was used in view of the theoretical relevance of the predictors to interrogation and false confession. As far as interrogation was concerned, the full multivariate model showed that CD was the single best predictor (IRR = 3.5, 95 % CI 3.2–3.8), followed by offending behaviour (IRR = 2.2, 95 % CI 2.0–2.4) and ADHD-symptomatic (IRR = 1.8, 95 % CI 1.6–2.1). Meanwhile, for number of false confessions, similar effect sizes were found for ADHD (IRR = 2.0, 95 % CI 1.4–2.7), CD (IRR = 2.0, 95 % CI 1.6–2.6) and age group (IRR = 2.0, 95 % CI 1.7–2.5; Note: IRR was inverted to reflect “risk” association).

**Table 3:** Summary of negative binomial regressions for interrogations and false confessions

Explanatory variables	*B* (SE)	*z*	IRR (95 % CI)
Interrogations^a^			
Age group (≥17 years)	0.64 (0.04)	15.4	1.9 (1.8–2.1)**
Gender	−0.80 (0.04)	−18.5	0.5 (0.4–0.5)**
Conduct disorder	1.25 (0.05)	25.8	3.5 (3.2–3.8)**
Offending behaviour	0.79 (0.05)	17.4	2.2 (2.0–2.4)**
ADHD-symptomatic	0.59 (0.07)	7.9	1.8 (1.6–2.1)**
False confessions^b^			
Age group (≥17 years)	−0.74 (0.12)	−6.2	0.5 (0.4–0.6)**
Gender	−0.38 (0.13)	−3.0	0.7 (0.5–0.9)*
Conduct disorder	0.71 (0.12)	5.8	2.0 (1.6 − 2.6)**
Offending behaviour	0.59 (0.12)	4.9	1.8 (1.4–2.3)**
ADHD-symptomatic	0.67 (0.16)	4.3	2.0 (1.4–2.7)**

* *p* < 0.01; ** *p* < 0.001

^a^LR test, *χ* ^2^ (1) = 1595.1, *p* < 0.001

^b^LR test, *χ* ^2^ (1) = 372.8, *p* < 0.001

### ADHD vulnerability status, police interrogation and false confession

The ADHD status of the 21,330 participants (where complete data were available—data were missing for 896 participants or 4 %) was categorised using the hierarchy of presentation according to severity of symptoms. Table 4 shows that both police interrogation and false confession were linearly related to ADHD status with a medium effect size (Cramer’s *V* = 0.15 and 0.22 for interrogation and false confession, retrospectively). Only 12.4 % of those who were not symptomatic and not on medication reported having been interrogated in contrast to 48.5 % of those who were medicated and symptomatic. With regard to false confession, the respective percentages were 10.8 and 40.2 %.

**Table 4:** Rate of interrogation and false confession across the four ‘diagnostic’ groups

Four groups	Interrogated *N* (%)	Not interrogated *N* (%)	False confession *N* (%)	No false confession *N* (%)
Not symptomatic and not medicated	2380 (12.4)	16,854 (87.6)	254 (10.8)	2105 (89.2)
Symptomatic and not medicated	249 (29.4)	599 (70.6)	57 (23.2)	189 (76.8)
Not symptomatic and medicated	207 (27.4)	548 (72.6)	66 (32.2)	139 (67.8)
Symptomatic and medicated	83 (48.5)	88 (51.5)	33 (40.2)	49 (59.8)
	*χ* ^2^ = 493.71*; *df* = 3, Cramer’s *V* = 0.15		*χ* ^2^ = 139.39*; *df* = 3, Cramer’s *V* = 0.22	

* *p* < 0.001

**Fig. 1:**
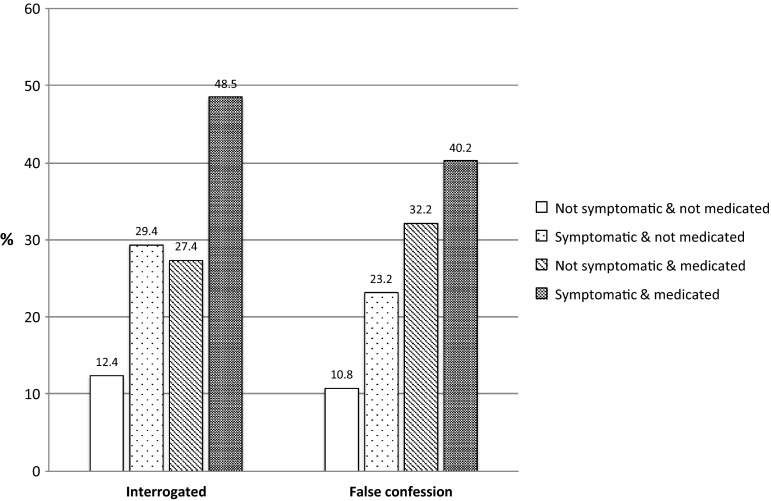
Proportion of false confession and interrogations in relation to symptom and medication status

To aid interpretation, the proportions of interrogations and false confessions in relation to severity are depicted graphically in Fig. 1. The severity of symptoms related to being on ADHD medication and still symptomatic was more linearly related to false confessions than interrogations. Meanwhile, interrogated participants who were symptomatic and on medication were disproportionately high when contrasted with those who were either symptomatic, or on medication only.

There was a strong relationship (large effect size) between the severity group classification and CD (*χ*^2^ = 1595.12, *p* < 0.001, Cramer’s *V* = 0.275), with CD being most common among the severity group 4 (64.8 %) and lowest among severity group 1 (11.4 %). There was a significant difference between those who were medicated and symptomatic versus those medicated and non-symptomatic with the former being more likely to have CD (*χ*^2^ = 89.22; *df* = 1, Cramer’s *V* = 0.307, *p* < 0.001, OR = 4.9, 95 % CI 3.5–6.9).

A similar pattern was found with respect to OB during the previous 12 months (medium effect size) (*χ*^2^ = 409.24, *df* = 3, Cramer’s *V* = 0.140), with the OB being most common among the severity group 4 (50.0 %) and lowest among severity group 1 (18.4 %). There was a significant difference between those who were medicated and symptomatic versus those medicated and non-symptomatic with the former being more likely to have offended (*χ*^2^ = 27.4, *df* = 1, Cramer’s *V* = 0.173, *p* < 0.001, OR = 2.4, 95 % CI 1.7–3.4).

## Discussion

The hypotheses related to both age and gender were supported. Participants in the older age group were twice more likely to have been interrogated than the younger group, but the younger participants were twice more likely to give a false confession when interrogated (both medium effect size). These findings add substantially to the literature and reinforce the preliminary findings of Gudjonsson et al.[6] by clearly demonstrating that younger youth (14–16) are considerably more vulnerable to giving false confessions than older youth (17–24). This may be due to the relative immaturity of the younger age group and their difficulties in coping with interrogative pressure [4, 10].

Males were more likely than females to report an interrogation and a false confession. The effect size was larger with regard to interrogations than false confessions (medium versus small effect size). This suggests that gender is good predictor of whom the police bring in for interrogation; this most likely relates to the higher level of CD and OB. Gender was a less powerful predictor of false confessions than interrogation, but the current study demonstrated that there was some relationship with the susceptibility to give false confessions. This merits further research, particularly as miscarriages of justice due to unreliable confessions predominantly involve males [32].

As hypothesised, there were differences in the relative contribution of CD and ADHD in relation to interrogation and false confessions and the severity of ADHD was a further contributory vulnerability factor. A screening diagnosis of CD increased the likelihood of having been interrogated 5.9 times and the likelihood of a false confession 2.6 times. The corresponding ORs for ADHD-combined were 4.3 and 3.7, respectively. The ORs for ADHD were lower (i.e. 3.1 and 2.6) when including the larger group of participants who met the screening criteria for ADHD-symptomatic. This suggests that the severity of ADHD in terms meeting screening criteria for both inattention and hyperactivity/impulsivity, referred to as the combined type, substantially increases the risk of being brought to a police station for interrogation and giving a false confession. The great majority of these comorbid young persons (76.8 %) were not being medicated for their condition at the time of the data collection.

NBR regressions showed that age, gender, conduct disorder, offending, and ADHD-symptomatic were all significant predictors of number of interrogations and false confessions. CD made the largest single contribution to the variance in police interrogations and CD and ADHD-symptomatic to false confessions. The findings suggest that CD is likely to bring young people to the attention of the police, but when interrogated their ADHD symptoms presents an additional vulnerability to giving a false confessions. This is consistent with research into false confessions among adult prisoners with ADHD [23]. The relative contribution of ADHD to interrogations and false confession in the regression model does not reflect the importance of the severity of the condition, which is well illustrated in Tables 1 and 2, because we included the more commonly used ADHD-symptomatic group rather than the much smaller combined type and those currently on medication.

The contribution to both police interrogation and false confession was also strongly related to our second measure of severity of the ADHD condition with those who were currently symptomatic and on medication (i.e. severity group 4) being most commonly interrogated (48.5 %) and reporting a history of a false confession (40.2 %). This vulnerability group, whose medication may be ineffective in reducing their ADHD symptoms, had the highest co-comorbidity with CD (64.8 %) and OB (50.0 %). They resembled the ADHD participants followed up by Satterfield et al. [20] and Langley et al. [21] who responded poorly to stimulant medication and had a high level of CD comorbidity, police contact, and offending. Young people with severe and untreated ADHD are also likely to be at the greatest risk of substance misuse [33] and re-offending [34, 35]. Even though this particular vulnerability group formed a very small part of the overall sample (<1 %), it is undoubtedly a group that requires the most urgent intervention to prevent future antisocial behaviour and persistent criminal trajectory. The overall effect of the severity of the ADHD condition was stronger for false confessions (Cramer’s *V* = 0.22) than interrogation (Cramer’s *V* = 0.15) and was more linearly related to the four group classification (see Fig. 1), suggesting a more direct relationship with false confessions.

Taken together, the two different measures of ADHD severity used in the current study, which only overlapped in a minority of cases, provide evidence that the severity of ADHD is an important risk factor in relation to both interrogation and false confession in addition to young persons being merely symptomatic in accordance with diagnostic screening criteria.

The strength of the study is its representative sample of a large national population; there were approximately similar number of participants in both groups and for the two genders. This made it possible to calculate and control for both age and gender differences in relation to interrogation experience and false confessions. In addition, the large sample size enabled the researchers to investigate the relationship of the combination of ADHD symptoms and medication status to both interrogation and false confessions, which has never been done before. The study was limited from its reliance on self-reported data as corroboration of responses was not obtained. Second, the current findings do not provide a complete explanation of factors related to false confession as this is likely to be complex. The false confession or false denial of an offence much depends on the situational context (e.g. the nature and duration of the interrogation, what suits the suspect at a given time) [22], as well as personality and health factors that were not measured in the current study [36].Third, as far as ADHD medication is concerned, it is a limitation that the participants were not asked about medication adherence, which is often a problem with children and adolescents diagnosed with ADHD [34, 37] and likely to be mediated by CD [38]. In Lichtenstein et al.’s [39] landmark study, medication was determined by the prescriptions issued, but there was no data available with regard to adherence. In spite of this, there were 32 and 41 % drop in official offending rate for males and females, respectively, whilst being prescribed ADHD medication. This suggests that ADHD medication may reduce offending and the potential mediators of adherence to medication and comorbid CD should be investigated in future. Multimodal interventions are likely to improve the treatment effect [40] suggesting that medication should be supplemented by programmes developed for this population such as R&R2 [41, 42]. Importantly, the findings of the present study raise the possibility that appropriate treatment may reduce the risk of a miscarriage of justice.

In summary, this large national epidemiological study adds to our understanding about the relative importance of ADHD, CD and OB with regard to police interrogation and false confessions. Fourteen percent of the participants reported having been subject to police interrogation and the study highlighted the important contribution of the severity of the ADHD condition and need for effective treatment, together with the need for support to be put in place to ensure fair due process and prevent miscarriages of justice.

## References

[1] Drizin SA, Leo RA (2004). The problem of false confessions in the post-DNA world. N C Law Rev.

[2] Garratt BL (2011). Convincing the innocent.

[3] Gudjonsson GH, Lassiter GD, Meissner CA (2010). The psychology of false confessions: A review of the current evidence. Police interrogations and false confessions.

[4] Kassin SM, Drizin SA, Grisso T, Gudjonsson GH, Leo RA, Redlich AD (2010). Police-induced confessions: risk factors and recommendations. Law Hum Behav.

[5] Redlich AD (2007). Double jeopardy in the interrogation room for youths with mental illness. Am Psychol.

[6] Gudjonsson GH, Sigurdsson JF, Asgeirsdottir BB, Sigfusdottir ID (2006). Custodial interrogation, false confession and individual differences. A national study among Icelandic youth. Personal Individ Differ.

[7] Gudjonsson GH, Sigurdsson JF, Sigfusdottir ID (2009). Interrogations and false confessions among adolescents in seven countries in Europe. What background and psychological factors best discriminate between false confessors and non-false confessors?. Psychol Crime Law.

[8] Sigurdsson JF, Gudjonsson GH (2001). False confessions: The relative importance of psychological, criminological and substance abuse variables. Psychol Crime Law.

[9] Young S, Moss D, Sedgwick O, Fridman M, Hodgkins P (2014) A meta-analysis of the prevalence of attention deficit hyperactivity disorder in incarcerated populations. Psychol Med. doi:10.1017/S0033291714000762 10.1017/S0033291714000762 PMC4301200 25066071

[10] Gudjonsson GH (2003). The psychology of interrogations and confessions. A handbook.

[11] Gudjonsson GH, Sigurdsson JF, Sigfusdottir ID, Young S (2012). False confessions to police and their relationship with conduct disorder, ADHD, and life adversity. Personal Individ Differ.

[12] Waschbusch DA (2002). A meta-analytic examination of comorbid hyperactive-impulsive-attention problems and conduct problems. Psychol Bull.

[13] Young S, Hopkin G, Perkins D, Farr C, Doidge A, Gudjonsson G (2013). A controlled trial of a cognitive skills program for personality-disordered offenders. J Atten Disord.

[14] Thapar A, Harrington R, McGuffin P (2001). Examining the comorbidity of ADHD-related behaviours and conduct problems using a twin study design. Br J Psychiatry.

[15] Young S, Gudjonsson GH (2006). ADHD symptomatology and its relationship with emotional, social and delinquency problems. Psychol Crime Law.

[16] Lynam DR (1996). Early identification of chronic offenders: who is the fledgling psychopath?. Psychol Bull.

[17] Gudjonsson GH, Sigurdsson JF, Sigfusdottir ID, Young S (2014). A national epidemiological study of offending and its relationship with ADHD symptoms and associated risk factors. J Atten Disord.

[18] Barkley RA, Murphy KR, Fischer M (2008). ADHD in adults. What the science says.

[19] Loeber R, Burke JD, Lahey BB, Winters A, Zera M (2000). Oppositional defiant and conduct disorder: a review of the past 10 years, part I. J Am Acad Child Adolesc Psychiatry.

[20] Satterfield JH, Faller KJ, Crinella FM, Schell AM, Swanson JM, Homer LD (2007). A 30-year prospective follow-up study of hyperactive boys with conduct problems: adult criminality. J Am Acad Child Adolesc Psychiatry.

[21] Langley K, Fowler T, Ford T, Thapar AK, van den Bree M, Harold G, Owen MJ, O’Donovan MC, Thapar A (2010). Adolescent clinical outcomes for young people with attention-deficit hyperactivity disorder. Br J Psychiatry.

[22] Gudjonsson GH, Sigurdsson JF, Bragason OO, Einarsson E, Valdimarsdottir EB (2004). Confessions and denials and the relationship with personality. Legal Criminol Psychol.

[23] Gudjonsson GH, Sigurdsson JF, Bragason OO, Newton AK, Einarsson E (2008). Interrogative suggestibility, compliance and false confessions among prisoners and their relationship with attention deficit hyperactivity disorder (ADHD) symptoms. Psychol Med.

[24] Gudjonsson GH, Sigurdsson JF, Einarsson E, Bragason OO, Newton AK (2010). Inattention, hyperactivity/impulsivity and antisocial personality disorder: which is the best predictor of false confessions?. Personal Individ Differ.

[25] Medford S, Gudjonsson GH, Pearse J (2003). The efficacy of the appropiate adult safeguard during police interviewing. Legal Criminol Psychol.

[26] Barkley RA (1998). Attention deficit/hyperactivity disorder: a handbook for diagnosis and treatment.

[27] Gudjonsson GH, Sigurdsson JF, Sigfusdottir ID, Asgeirsdottir BB (2008). False confessions and individual differences. The importance of victimisation among youth. Personal Individ Differ.

[28] Lewinsohn PM, Rohde P, Farrington DP (2000). The OADP-CDS: a brief screener for adolescent conduct disorder. J Am Acad Child Adolesc Psychiatry.

[29] Sigfusdottir ID, Farkas G, Silver E (2004). The role of depressed mood and anger in the relationship between family conflict and delinquent behavior. J Youth Adolesc.

[30] Long JS, Freese J (2006). Regression models for categorical dependent variables using Stata.

[31] StataCorp (2013). Stata statistical software: release 13.

[32] Gudjonsson GH (2010). Invited article. Psychological vulnerabilities during police interviews. Why are they important?. Legal Criminol Psychol.

[33] Gonzalez RA, Velez-Pastrana MC, Ruiz Varcarcel JJ, Levin FR, Albizu-Garcia CE (2015). Childhood ADHD symptoms are associated with lifetime and current illicit substance-use disorders and in-site health risk behaviors in a representative sample of Latino prison inmates. J Atten Disord.

[34] Young S, Wells J, Gudjonsson GH (2011). Predictors of offending among prisoners: the role of attention-deficit hyperactivity disorder and substance use. J Psychopharmacol.

[35] Gonzalez RA, Gudjonsson GH, Wells J, Young S (2013). The role of emotional distress and ADHD on institutional behavioral disturbance and recidivism among offenders. J Atten Disord.

[36] Gudjonsson GH, St-Yves M (2014). Mental vulnerabilities and false confession. Investigative interviewing. The essentials.

[37] Adler LD, Nierenberg AA (2010). Review of medication adherence in children and adults with ADHD. Postgrad Med.

[38] Thiruchelvam D, Charach A, Schachar RJ (2001). Moderators and mediators of long-term adherence to stimulant treatment in children with ADHD. J Am Acad Child Adolesc Psychiatry.

[39] Lichtenstein P, Halldner L, Zetterqvist J, Sjolander A, Serlachius E, Fazel S, Langstrom N, Larsson H (2012). Medication for attention deficit–hyperactivity disorder and criminality. N Engl J Med.

[40] Arnold LE, Hodgkins P, Kahle J, Madhoo M, Kewley G (2015). Long-term outcomes of ADHD: academic achievement and performance. J Atten Disord.

[41] Rees-Jones A, Gudjonsson G, Young S (2012). A multi-site controlled trial of a cognitive skills program for mentally disordered offenders. BMC psychiatry.

[42] Young S, Goodwin EJ, Sedgwick O, Gudjonsson GH (2013). The effectiveness of police custody assessments in identifying suspects with intellectual disabilities and attention deficit hyperactivity disorder. BMC Med.

